# Effects of leptin on the viability of human ovarian cancer cells and changes in cytokine expression levels

**DOI:** 10.7717/peerj.15246

**Published:** 2023-04-20

**Authors:** Fatih Dincer, Harika Atmaca, Levent Akman, Latife Merve Oktay, Burcak Karaca, Mustafa Cosan Terek

**Affiliations:** 1 Divison of Gynecologic Oncology, Health Sciences University İzmir Tepecik Education and Research Hospital, Health Sciences University, Izmir, Turkey; 2 Department of Biology, Faculty of Science and Letters, Manisa Celal Bayar University, Manisa, Turkey; 3 Department of Obstetrics and Gynecology, Divison of Gynecologic Oncology, Ege University, Bayraklı, İzmir, Turkey; 4 Department of Medical Biology, Medicine Faculty, Ege University, Bayraklı, İzmir, Turkey; 5 Department of Medical Oncology, Tulay Aktas Oncology Hospital, Ege University, Bayraklı, İzmir, Turkey

**Keywords:** Obesity, Leptin, Ovarian cancer

## Abstract

**Background:**

Obesity is associated with increased mortality among ovarian cancer and is a poor prognostic factor. There are significant links between the leptin hormone, a product of the obesity gene, and the development of ovarian cancer. Leptin is a vital hormone-like cytokine secreted from adipose tissue and is mainly involved in the maintenance of energy homeostasis. It regulates several intracellular signaling pathways and also interacts with various hormones and energy regulators. It acts as a growth factor by stimulating cell proliferation and differentiation and in this way contributes to cancer cell development. The aim of the study was to investigate the effects of leptin on human ovarian cancer cells.

**Methods:**

In this study, the effects of increasing the concentration of leptin were investigated on the cell viability of OVCAR-3 and MDAH-2774 ovarian cancer lines by MTT assay. Moreover, to elucidate the molecular mechanisms of leptin in ovarian cancer cells, changes in the expression levels of 80 cytokines were evaluated after leptin treatment *via* a human cytokine antibody array.

**Results:**

Leptin increases the proliferation of both ovarian cancer cell lines. IL-1 level was increased in OVCAR-3 cells and TGF-β level was increased in MDAH-2774 cells after leptin treatment. A decrease in IL-2, MCP-2/CCL8 and MCP-3/CCL7 levels was detected in both ovarian cancer cell lines with leptin administration. An increase in IL-3 and IL-10 expressions, insulin-like growth factor binding proteins (IGFBP) IGFBP-1, IGFBP-2 and IGFBP-3 levels were detected in both ovarian cancer cell lines with leptin administration. In conclusion; leptin has a proliferative effect on human ovarian cancer cell lines and affects different cytokines in different types of ovarian cancer cells.

## Introduction

Ovarian cancer (OC) is the deadliest among gynecological cancers since it does not give any symptoms in the early period and there is no effective screening method for early diagnosis (Parker et al., 2009). OC is a heterogeneous disease; it originates from different gynecological tissues, has different clinical characteristics, and therefore gives different responses to treatment methods. In OC, the 5-year survival rate is about 30%. The reason for the high mortality of the disease is that it does not give any symptoms in the early period and there is no effective screening method for early diagnosis (Parker et al., 2009; Longuespée et al., 2012).

Nowadays, the 5-year survival rate has increased from 37% to 45% in ovarian cancer patients, thanks to successful cytoreductive surgery, platinum components and chemotherapy combined with taxanes (Romero & Bast, 2012). After surgical removal of tumor tissues and chemotherapy, in 60–70% of patients, the disease recurs and resistance to platinum-based chemotherapy develops (Gao et al., 2013). Therefore, novel drugs and treatment options that are effective in the treatment of ovarian cancer are needed.

Obesity is associated with increased mortality among ovarian cancer and is a poor prognostic factor (Tworoger & Huang, 2016). In the literature, there are significant links between the leptin hormone, a product of the obesity gene, and the development of ovarian cancer (Liu et al., 2015; Howe et al., 2013). Leptin is a vital hormone-like cytokine secreted from adipose tissue and is mainly involved in the maintenance of energy homeostasis (Ray, 2012). It regulates several intracellular signaling pathways including Janus kinase (JAK)-signal transducer and activator of transcription (STAT), phosphatidylinositol-3-kinases (PI3K), and mitogen-activated protein kinase (MAPK) (Dutta et al., 2012). It also interacts with various hormones and energy regulators such as insulin, glucagon, insulin-like growth factor, growth hormone, glucocorticoids, cytokines, and metabolites (Margetic et al., 2002; Divella et al., 2016). It acts as a growth factor by stimulating cell proliferation and differentiation and in this way contributes to cancer cell development (Dutta et al., 2012; Ray & Cleary, 2017). Revealing the mechanisms of leptin causing tumor formation and suppression of these mechanisms can be considered as a treatment strategy.

In this study, the effects of leptin were investigated on the viability of human OVCAR-3 and MDAH-2774 ovarian cancer cell lines with different genotypes. Moreover, changes in the secretion of cytokines after leptin treatment were evaluated *via* a human cytokine antibody array.

## Materials and Methods

### Reagents

Human recombinant Leptin was purchased from PeproTech (USA) and dissolved in phosphate-buffered saline (1 mg/mL), according to the manufacturer’s protocols. All the cell culture supplies were obtained from Sigma Aldrich (UK).

### Cells and culture

OVCAR-3 and MDAH-2774 cells were purchased from Interlab Cell Line Collection (ICLC, Genova, Italy). OVCAR-3 cells were cultured in RPMI 1640 supplemented with 10% fetal bovine serum, 1% L-glutamine, 1% penicillin-streptomycin in 75 cm^2^ polystyrene flasks and maintained at 37 °C in a humidified atmosphere with 5% CO_2_. MDAH-2774 cells were maintained in DMEM supplemented with 10% FBS, 1% penicillin/streptomycin and 1% L-glutamine.

### XTT cell viability assay

The cells after 24 and 48 h serum starvation, were treated with increasing concentrations of human recombinant leptin (0.5–400 ng/mL) for 24, 48 and 72 h. The effect of leptin treatment on cell viability was measured by XTT assay (Sigma-Aldrich, St. Louis, MO, USA). OVCAR-3 and MDAH-2774 cells were cultured on 96-well plates at a concentration of 1 × 10^4^ cells per well. After leptin treatment for indicated periods, 100 µL XTT solution was added to each well and, after 4 h incubation the absorbance of each well was measured at 570 nm on a microplate reader (Beckman Coulter, multimode detector, USA).

### Cytokine array

Changes in the cytokine levels were assessed by a Human Cytokine Antibody Array (Raybiotech, GA, USA) according to the instruction manual. The list of cytokines assessed is shown in Table 1. Both cancer cells were cultured in 6-well plates at a concentration of 2 × 10^6^ cells per well. Then, OVCAR-3 cells were starved for 48 h and then treated with 50 ng/mL Leptin for 48 h. However, MDAH-2774 cells were starved for 24 h and then treated with 50 ng/mL Leptin for 72 h. Briefly, array membranes were blocked at room temperature for 30 min. Cells were lysed with lysis buffer and lysates were incubated on the blocked membranes overnight at 4 °C. After incubation, membranes were washed twice with the washing solution and incubated with a biotinylated antibody followed for 1 h at room temperature. After another washing step, membranes were incubated with horseradish peroxidase-conjugated streptavidin for 1 h. The forming signals were detected using chemiluminescence in the Kodak® Gel Logic 1500 imaging system (Carestream Molecular Imaging, New Haven, CT, USA). The spots were quantified by Koadarray® 2.6 software.

**Table 1:** Effects of Leptin on cell viability of OVCAR-3 cells.

OVCAR-3						
Leptin (ng/ml)	24 h serum starvation			48 h serum starvation		
	24 h	48 h	72 h	24 h	48 h	72 h
0.5	130.5*	80.9	78.5	141.7*	8.5	78.5
5	129.2*	50.3	64.3	113.1	73.7	86.7
25	137.5*	89.6	83.4	142.9*	110.7	62.7
50	148.9*	76.6	127.4	187.4*	117.7	71.8
100	179.2*	83.4	96.3	147.6*	140.9	82.9
200	125.9*	91.9	122.6	123.8*	89.5	65.7
400	139.2*	104.2	119.4	120.9*	107.1	62.1

**Note:**

* *p* < 0.05.

### Statistical analysis

Statistical analysis was performed using Graphpad Prism 5.0 software. Statistical analysis was performed by one-way analysis of variance followed by *Dunnett’s t-test* for multiple comparisons, where *p* < 0.05 was assumed as statistically significant. All samples were analyzed in triplicates and the results were expressed as the mean ± (SD).

## Results

### Effects of Leptin on cell viability of OVCAR-3 and MDAH-2774 cells

After 24 h serum starvation, OVCAR-3 cells were treated with increasing concentrations of leptin (0.5–400 ng/mL) and cell viability was evaluated at 24, 48 and 72 time points (Table 1). After 24 h leptin exposure, cell viability was increased even at the highest leptin concentrations as untreated compared to the control group. However, after 48 h of leptin exposure, the percentage of cell viability was reduced slightly at 0.5–200 ng/mL concentrations (*p* < 0.05). Similar results were also obtained at 72 h leptin exposure; cell viability was reduced slightly by 0.5–25 ng/mL concentrations at this time point (*p* < 0.05) (Table 1).

Then, OVCAR-3 cells were starved for 48 h and treated with increasing concentrations of leptin (0.5–400 ng/mL) and cell viability was calculated at 24, 48, and 72 h (Table 1). Following 24 h leptin exposure, cell viability was increased by all tested concentrations of leptin. But, after 48 and 72 h leptin exposure cell viability decreased slightly.

The same experimental design was applied to MDAH-2774 cells; after 24 h serum starvation, MDAH-2774 cells were treated with increasing concentrations of leptin (0.5–400 ng/mL) and cell viability was evaluated at 24, 48 and 72 h (Table 2). Different from OVCAR-3 cells, cell viability was increased at all tested concentrations in MDAH-2774 cells at 24, 48 and 72 h time points (Table 2).

**Table 2:** Effects of Leptin on cell viability of MDAH-2774 cells.

MDAH-2774						
Leptin (ng/ml)	24 h serum starvation			48 h serum starvation		
	24 h	48 h	72 h	24 h	48 h	72 h
0.5	109.2	107.1	93.8	101.3	102.1	92.8
5	103.7	108.9	112.9	102.9	107.8	99.1
25	99.4	120.8*	127.1*	105.7	114.3	101.8
50	113.5	105.5	122.4*	100.6	120.2*	123.7*
100	96.2	107.2	123.7*	104.6	117.3	105.9
200	116.1	92.6	111.7	101.6	109.1	89.5
400	112.6	119.6*	108.8	116.2	108.3	93.6

**Note:**

* *p* < 0.05.

After 48 h serum starvation of MDAH-2774 cells, and treatment with increasing concentrations of leptin for 24 h, cell viability was not significantly decreased (Table 2). Similar results were obtained after 48 h exposure to leptin; however, at 72 h, cell viability reduced slightly (*p* > 0.05) (Table 2).

### Changes in the cytokine levels after leptin exposure in ovarian cancer cells

The changes in the number of cytokines measured by the cytokine antibody array method and show statistically significant change are given in Tables 3 and 4. OVCAR-3 and MDAH-2774 cells showed different cytokine profiles after treatment with leptin. In OVCAR-3 cells, 50 ng/mL leptin exposure resulted in significant changes in the expression levels of interleukin (IL) family members such as IL-1 (+2.9), IL-2 (−2.1), IL-3 (−1.8), IL-5 (−2.4), IL-8 (+1.6), IL-10 (+2.3), IL-12 (+1.7) and IL-13 (+1.7) (Table 3). There were also significant changes in the expression levels of growth factors such as EGF (+1,9), PDGF-BB (+2,1), FGF-6 (+1,8), PLGF (+2.9) and TGF beta 3 (+6.6) (Table 3).

**Table 3:** Changes in the cytokine levels after leptin exposure in OVCAR-3 cells.

Cytokine	Amount of change	± Std
GM-CSF	3.1	0.168
Gro a/b/g	1.95	0.556
Gro alfa (CXCL1)	2.2	0.227
IL-1 (IL-1 F1)	2.9	0.1056
IL-2	−2.1	0.436
IL-3	−1.8	0.82
IL-5	−2.4	0.336
IL-8 (CXCL 8)	1.6	0.92
IL-10	2.3	0.362
IL-12 (p40/p70)	1.7	0.8792
IL-13	1.7	0.978
MIG (CXCL9)	−2.3	0.2312
MIP-1 delta	−2.1	0.1228
TNF beta (TNFSF 1B)	−1.8	0.254
EGF	1.9	0.718
PDGF-BB	2.1	0.1562
BDNF	1.7	0.887
BLC (CXCL 13)	1.8	0.689
Ck beta 8-1 (CCL23)	−1.8	0.453
Eotaxin-1 (CCL11)	1.7	0.743
FGF-6	1.8	0.2256
GCP-2 (CXCL6)	1.7	0.357
IGFBP-1	1.9	0.649
IGFBP-3	2.4	0.1632
LIGHT (TNFSF14)	1.6	0.489
MCP-4 (CCL13)	3.7	0.2215
NT-4	3.3	0.456
PLGF	2.9	0.327
TGF beta 3	6.6	0.2522

**Table 4:** Changes in the cytokine levels after leptin exposure in MDAH-2774 cells.

Cytokine	Amount of change	± Std
ENA-78 (CXCL5)	−3.6	0.2316
G-CSF	−2.1	0.4459
IL-2	−2.6	0.3625
IL-3	−2.5	0.4479
IL-10	−2.3	0.3345
IFN-gamma	−1.9	0.829
MCP-2 (CCL8)	−2.4	0.7523
MCP-3 (CCL7)	−2.2	0.8432
MIG (CXCL9)	8.8	0.1223
PDGF-BB	−2.2	0.2249
BDNF	−1.6	0.7563
Eotaxin-1 (CCL11)	−2.3	0.3648
FGF-4	1.7	0.9231
FLT-3 ligand	−2.1	0.6456
IGFBP-2	−2	0.561
IL-16	1.8	0.4793
MCP-4 (CCL13)	−1.7	0.862
NAP-2 (CXCL7)	−1.7	0.635
OPN (SPP1)	−1.9	0.4923
PLGF	−1.9	0.9712
TGF beta 3	2	0.2812

In MDAH-2774 cells, 50 ng/mL leptin exposure also resulted in significant changes in the expression levels of interleukin (IL) family members such as IL-2 (−2.6), IL-3 (−2.5), IL-10 (−2.3) and IL-16 (+1.8). Similar to the OVCAR-3 cells, there were significant changes in the expression levels of growth factors such as PDGF-BB (−2.2), FGF-4 (+1.7), PLGF (−1.9) and TGF beta 3 (+2.0) (Table 4).

## Discussion

Obesity, whose incidence is increasing rapidly, is an important health problem for developed countries. It is considered a risk factor for prostate, breast, esophagus, colon and lung cancers as well as diabetes and cardiovascular diseases (Calle et al., 2003; Bray, 1999). Epidemiological studies show that obesity increases the incidence of ovarian cancer, increases survival rate and the risk of recurrence (Leitzmann et al., 2009; Schouten et al., 2008; Calle et al., 2003; Rodriguez et al., 2002; Pavelka et al., 2006). However, the molecular mechanisms underlying clinical observations have not been elucidated yet.

Leptin is a hormone secreted by adipocytes belonging to the helical cytokine family. It is a multifunctional peptide hormone playing role in a variety of biological activities, such as appetite regulation, bone formation, angiogenesis, initiation and modulation of autoimmune responses (Margetic et al., 2002; Anifandis et al., 2005). Leptin is known to play an important role in natural and acquired immunity (Friedman, 2009). The increase of leptin levels during infection or inflammation indicates the importance of leptin on the host’s response to inflammation. In addition to its important roles in metabolism, leptin is known to have a mitogenic effect in breast, prostate and gastrointestinal cancers. It has been shown to induce proliferation, migration, invasion and angiogenesis of cancer cells, which is thought to trigger aggressive cancer phenotype (Garofalo & Surmacz, 2006; Jardé et al., 2011).

The expression of leptin receptors in the ovary and the presence of leptin in the follicular fluid indicate that leptin has a role in ovarian functions (Choi et al., 2005). There are many studies investigating the possible role of leptin in ovarian cancer, but its molecular mechanisms have not been elucidated yet.

In this study, the effects of increasing concentration of leptin were investigated on cell viability of OVCAR-3 and MDAH-2774 ovarian cancer lines. Moreover, to elucidate the molecular mechanisms of leptin in ovarian cancer cells, changes in the expression levels of 80 cytokines were evaluated after leptin treatment.

From the results, it has been shown that leptin increases the proliferation of both ovarian cancer cell lines in a serum-starved medium. Similarly, Ptak, Kolaczkowska & Gregoraszczuk (2013) examined the effects of leptin on the proliferation of OVCAR-3 ovarian cancer cells in a dose-dependent manner and showed that leptin (2 ng/ml–100 ng/ml) increased the proliferation of cells. Uddin et al. (2009) examined the effects of leptin on the proliferation of MDAH-2774 cell lines and found that leptin had stimulating effects. In other *in vitro* studies, leptin has been shown to increase the viability of BG-1 ovarian cancer cells and inhibit apoptosis in SKOV-3 and MDAH-2774 ovarian cancer cells (Choi, Lee & Leung, 2011). In the study by Choi et al. (2005), it was determined that 1 and 10 ng/ml doses of leptin had no effect on the proliferation of BG-1 cells, which is the human ovarian cancer cell line, and 100 and 1,000 ng/mL doses increased proliferation. In the same study, the researchers found that leptin receptors were expressed in BG-1, OVCAR-3, SKOV-3 and IOSE-80PC cell lines, but not all cell lines responded to leptin in the same way. They found that leptin did not affect the proliferation of SKOV3 and IOSE-80PC ovarian cancer cell lines. This situation has been interpreted as an indication that leptin does not affect each cell in the same way, although there are leptin receptors.

Cytokines are proteins produced by almost all cells that stimulate proliferation, regulate cell differentiation and induce cell chemotaxis. They also regulate the expression of other cytokines. Leptin signaling is known to increase proinflammatory cytokine signaling. The expression and secretion of protein signals and factors such as TNFα and IL-6 have been shown to increase due to the increased amount of leptin in obese individuals (Hotamisligil, 2006; Rajala et al., 2003; Trayhurn, 2013).

Since adipose tissue is metabolically active and secretes estrogens, adipokines (such as leptin) and secrete cytokines, a tight connection has been established between obesity and cancer (Lumeng, Bodzin & Saltiel, 2007). Therefore, researchers are investigating the effects of leptin on cytokines in cancer cells. Alemán et al. (2002) examined leptin serum levels in non-small cell lung cancer patients and found that leptin levels were less than in healthy individuals. Besides, they found that the levels of CRP, ferritin, TNF-a, IL-6 are inversely proportional to the leptin level (Alemán et al., 2002). There are studies showing that high leptin or leptin receptor levels in primary ovarian tumors are associated with a poor prognosis (Uddin et al., 2009; Diaz, Karlan & Li, 2013). The effects of leptin on cytokines in ovarian cancer cells have not been investigated yet.

Here, we aimed to clarify the molecular mechanisms of leptin in ovarian cancer cells. For this purpose, changes in cytokines secreted from leptin-treated OVCAR-3 and MDAH-2774 ovarian cancer cells were investigated by a human cytokine antibody array. Significant changes were detected in secreted cytokines in OVCAR-3 and MDAH-2774 cells by leptin treatment. Kato et al. (2015) have shown that high leptin levels increase the release of various cytokines from cancer cells. Many cytokines have been identified in the acid fluids of cancer patients and these cytokines are known to be higher in obese patients (Kato et al., 2015). Among these cytokines, the interleukin (IL) family has complex immunomodulatory functions such as cell proliferation, differentiation, migration, and adhesion. It is known that IL-1 increases tumor growth and is secreted when cell metabolism changes, such as in obesity (Tack et al., 2012). IL-1 is also a proangiogenic factor that increases VEGF expression. It was determined that an increase of IL-1 was associated with leptin-mediated activation of JAK2/STAT, PKC, p38, MAPK/ERK1/2, PI-3K/AKT1 signaling pathways. For these reasons, the relationship between IL-1 and leptin is critical for tumor growth and angiogenesis (Mullen & Gonzalez-Perez, 2016). In our study, an increase in the level of IL-1 was detected in leptin-treated OVCAR-3 cells. IL-2, on the other hand, is a key molecule that plays a role in the regulation of mitogenic signals necessary for the differentiation of T and NK lymphocytes, the main tumor defender effectors. Therefore, it has attracted attention as a target molecule in cancer treatment and has been approved for metastatic melanoma treatments with metastatic renal carcinomas (Sim & Radvanyi, 2014). A decrease in IL-2 levels was detected in both ovarian cancer cell lines with leptin administration. In addition, with leptin administration, IL-3 and IL-10 expressions increased in both ovarian cancer cell lines. IL-3 is released into the tumor microenvironment to support the survival and proliferation of endothelial cells. It is known that IL-3, which is an endothelial differentiation factor, plays an important role in tumor vascularization (McBride et al., 1994). IL-10 is known as an immunosuppressive cytokine. There was a correlation between IL-10 and tumor growth. It was also associated with poor progression of cancer (Mannino et al., 2015). Matte et al. (2012) showed that patients with high IL-10 levels had a reduced lifespan (Struyf et al., 2009). Chemokines are molecules involved in inflammation and tumor development. Of these, MCP-2/CCL8 and MCP-3/CCL7 decreased as a result of leptin treatment in both ovarian cancer cell lines. MCP-2/CCL8 is a pluripotent chemokine that can interact with many cells such as leukocytes, NK cells, monocytes, and basophils, as it can bind to various receptors such as CCR1, CCR2, CCR3 and CCR5. MCP-3/CCL7 is known to suppress T lymphocytes and NK cells and also suppress tumor formation (Matte et al., 2012). MIG/CXCL9 is an anti-tumor response molecule by suppressing IL-12, which is considered an important target in ovarian cancer patients (Rainczuk et al., 2012).

By interacting with interferon (IFN) and its receptor, JAK family kinases are activated to regulate STAT molecules, phosphorylates and transcription. It has been shown that leptin supports the inflammatory response by working with IFN. It is known that IFN-γ inhibits tumor angiogenesis and increases MHC expression, which plays a role in tumor recognition (Fernández-Riejos et al., 2010). A reduction in this antiangiogenic molecule was detected in MDAH 2774 ovarian cancer cell line with leptin application.

TGF-β is another molecule that increases in MDAH 2774 cell line after leptin treatment. TGF-β is a molecule that has a dual role in tumor formation. It acts as a tumor suppressor in the cell cycle and apoptosis by providing homeostasis in the epithelium, endothelium, and hematopoietic cells in the early stages of tumor formation. TGF-β is an inflammatory cytokine that bridges leptin and inflammatory response since it modulates the release of leptin from adipose and tumor cells (Mullen & Gonzalez-Perez, 2016).

Insulin-like growth factor binding proteins (IGFBP) IGFBP-1, IGFBP-2 and IGFBP-3 were increased by leptin treatment in both ovarian cancer cells. They bind to IGFs and carry the cellular IGFs which act as an insulin hormone in the body. IGFBPs are produced by many cells and they were detected in many tissue fluids. They prevent IGFBP-1 and IGFBP-2 from binding by activating them by binding to IGF’s receptors. IGFBP-3 is the most abundant protein in serum, suppresses the antiapoptotic effects of IGF and regulates its mitogenic activities. The high level of IGFBP-3 in serum means a high risk for many types of cancer, including ovarian cancer (Fernández-Riejos et al., 2010).

Studies on which biological mechanisms and how obesity affects cancer risk; show that there may be different mechanisms for different types of cancer. Yet, for each type of cancer, the main mechanisms causing these cancers have not been precisely explained. In this study, for the first time in the literature, the effects of leptin on the levels of cytokines in ovarian cancer cells have been revealed. It was shown that leptin has a proliferative effect on human ovarian cancer cell lines and affects different cytokines in different types of ovarian cancer cells.

## Supplemental Information

10.7717/peerj.15246/supp-1 Supplemental Information 1 Cytokin array layout.

10.7717/peerj.15246/supp-2 Supplemental Information 2 Cytokin array datasheet.

10.7717/peerj.15246/supp-3 Supplemental Information 3 MDAH and leptin treatment.

10.7717/peerj.15246/supp-4 Supplemental Information 4 MDAH cell control group.

10.7717/peerj.15246/supp-5 Supplemental Information 5 Leptin treatment in ovcar cell line; cytokin array results.

10.7717/peerj.15246/supp-6 Supplemental Information 6 Leptin treatment in mdah cell line; cytokin array results.

10.7717/peerj.15246/supp-7 Supplemental Information 7 Ovcar 3 and leptin treatment.

10.7717/peerj.15246/supp-8 Supplemental Information 8 OVCAR 3 cell control group.

## Additional Information and Declarations

### Microarray Data Deposition

The following information was supplied regarding the deposition of microarray data: Raybiotech, GA, USA.

### Data Availability

The following information was supplied regarding data availability: The raw measurements are available in the Supplemental Files.
