# Supplementary material for: Effects of leptin on the viability of human ovarian cancer cells and changes in cytokine expression levels
Source: PeerJ. 2023 Apr 20;11:e15246. doi: 10.7717/peerj.15246 (PMC10122840; doi:10.7717/peerj.15246)
Supplement: Supplemental Information 1 [file peerj-11-15246-s001.docx]

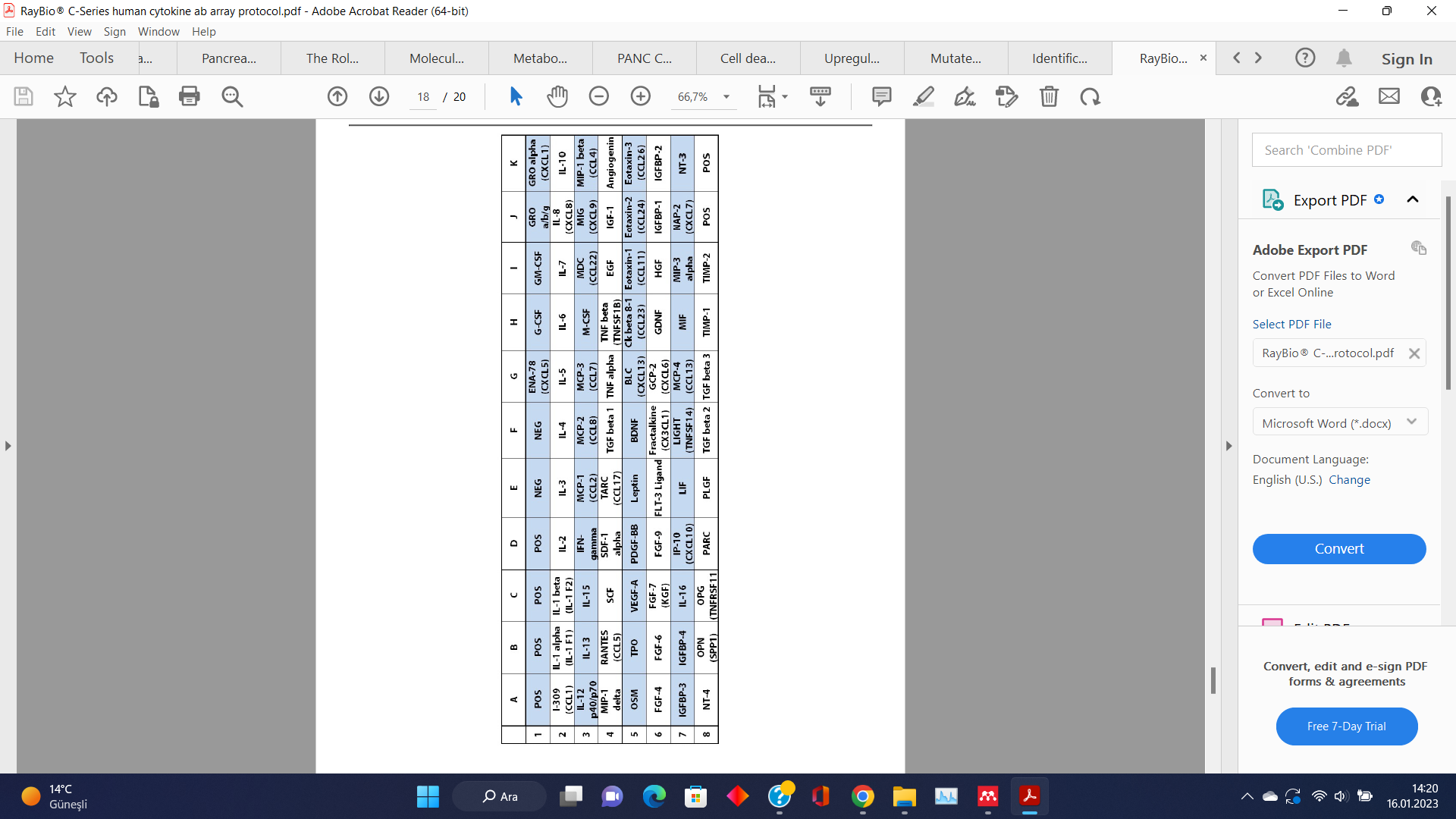


| **Sp_X** | **Sp_Y** | **Sitokin Adı** |
| --- | --- | --- |
| 1 | 1 | Pozitif kontrol |
| 2 | 1 | Pozitif kontrol |
| 3 | 1 | Pozitif kontrol |
| 4 | 1 | Pozitif kontrol |
| 5 | 1 | Negatif kontrol |
| 6 | 1 | Negatif kontrol |
| 7 | 1 | ENA-78 (CXCL5) |
| 8 | 1 | G-CSF |
| 9 | 1 | GM-CSF |
| 10 | 1 | GRO a/b/g |
| 11 | 1 | GRO aplha (CXCL1) |
| 1 | 2 | CCL1 |
| 2 | 2 | IL-1 alpha |
| 3 | 2 | IL-1 beta |
| 4 | 2 | IL-2 |
| 5 | 2 | IL-3 |
| 6 | 2 | IL-4 |
| 7 | 2 | IL-5 |
| 8 | 2 | IL-6 |
| 9 | 2 | IL-7 |
| 10 | 2 | IL-8 |
| 11 | 3 | IL-10 |
| 1 | 3 | IL-12 |
| 2 | 3 | IL-13 |
| 3 | 3 | IL-15 |
| 4 | 3 | IFN-gamma |
| 5 | 3 | MCP-1 |
| 6 | 3 | MCP-2 |
| 7 | 3 | MCP-3 |
| 8 | 3 | M-CSF |
| 9 | 3 | MDC |
| 10 | 3 | MIG |
| 11 | 3 | MIP-1 beta |
| 1 | 4 | MIP-1 delta |
| 2 | 4 | RANTES |
| 3 | 4 | SCF |
| 4 | 4 | SDF-1 alpha |
| 5 | 4 | TARC |
| 6 | 4 | TGF beta1 |
| 7 | 4 | TNF alpha |
| 8 | 4 | TNF beta |
| 9 | 4 | EGF |
| 10 | 4 | IGF-1 |
| 11 | 4 | Angiogenin |
| 1 | 5 | OSM |
| 2 | 5 | TPP |
| 3 | 5 | VEGF-A |
| 4 | 5 | PDGF-BB |
| 5 | 5 | Leptin |
| 6 | 5 | BDNF |
| 7 | 5 | BLC |
| 8 | 5 | Ck-beta 8-1 |
| 9 | 5 | Eotaxin-1 |
| 10 | 5 | Eotaxin-2 |
| 11 | 5 | Eotaxin-3 |
| 1 | 6 | FGF-4 |
| 2 | 6 | FGF-6 |
| 3 | 6 | FGF-7 |
| 4 | 6 | FGF-9 |
| 5 | 6 | FLT-3 Ligand |
| 6 | 6 | CX3CL |
| 7 | 6 | GCP-2 |
| 8 | 6 | GDNF |
| 9 | 6 | HGF |
| 10 | 6 | IGFBP-1 |
| 11 | 6 | IGFBP-2 |
| 1 | 7 | IGFBP-3 |
| 2 | 7 | IGFBP-4 |
| 3 | 7 | IL-16 |
| 4 | 7 | IP-10 |
| 5 | 7 | LIF |
| 6 | 7 | TNFSF14 |
| 7 | 7 | MCP4 |
| 8 | 7 | MIF |
| 9 | 7 | MIP-3 |
| 10 | 7 | NAP-2 |
| 11 | 7 | NT-3 |
| 1 | 8 | NT-4 |
| 2 | 8 | OPN |
| 3 | 8 | OPG |
| 4 | 8 | PARC |
| 5 | 8 | PLGF |
| 6 | 8 | TGF beta 2 |
| 7 | 8 | TGF beta 3 |
| 8 | 8 | TIMP-1 |
| 9 | 8 | TIMP-2 |
| 10 | 8 | Pozitif kontrol |
| 11 | 8 | Pozitif kontrol |
