# Supplementary material for: Effects of leptin on the viability of human ovarian cancer cells and changes in cytokine expression levels
Source: PeerJ. 2023 Apr 20;11:e15246. doi: 10.7717/peerj.15246 (PMC10122840; doi:10.7717/peerj.15246)
Supplement: Supplemental Information 2 [file peerj-11-15246-s002.pdf]

RayBio® C-Series
Human Cytokine Antibody Array C5

For the semi-quantitative detection of 80 human proteins in serum, plasma, cell culture media, and other liquid sample types.

Patent Pending Technology

AAH-CYT-5-2 (2 Sample Kit)
AAH-CYT-5-4 (4 Sample Kit)
AAH-CYT-5-8 (8 Sample Kit)

User Manual
Last revised February 26, 2016

Caution:
Extraordinarily useful information enclosed

RayBiotech
The protein array pioneer

ISO 13485 Certified

3607 Parkway Lane, Suite 100
Norcross, GA 30092
Tel: 1-888-494-8555 (Toll Free) or 770-729-2992, Fax:770-206-2393
Web: www.RayBiotech.com, Email: info@raybiotech.com

Table of Contents

Section		Page #
I.	Introduction	3
II.	How It Works	5
III.	Components and Storage	6
IV.	Additional Materials Required	6
V.	Sample Tips and General Considerations<br>A. Sample Collection, Preparation and Storage<br>B. Sample Types and Recommended Dilutions/Amounts<br>C. Handling Membranes<br>D. Incubations and Washes	7<br>7<br>8<br>8<br>8
VI.	Chemiluminescence Detection Tips	9
VII.	Component Preparation	10
VIII.	Protocol<br>A. Blocking<br>B. Sample Incubation<br>C. First Wash<br>D. Biotinylated Antibody Cocktail Incubation<br>E. Second Wash<br>F. HRP-Streptavidin Incubation<br>G. Third Wash<br>H. Chemiluminescence Detection<br>I. Storage	11<br>11<br>11<br>12<br>12<br>12<br>12<br>13<br>13<br>14
IX.	Typical Results	14
X.	Interpreting the Results<br>A. Control Spots<br>B. Data Extraction<br>C. Data Analysis	15<br>15<br>15<br>16
XI.	Array Map	18
XII.	Troubleshooting Guide	19

Please read the entire manual carefully before starting your experiment

I. Introduction

New techniques such as cDNA microarrays have neabled us to analyze global gene expression[1-3]. However, almost all cell functions are executed by proteins, which cannot be studied simply through DNA and RNA techniques. Experimental analysis clearly shows disparity can exist between the relative expression levels of mRNA and their corresponding proteins[4]. Therefore, analysis of the proteomic profile is critical

The conventional approach to analyzing multiple protein expression levels has been to use 2-D SDS-PAGE coupled with mass spectrometry[5,6]. However, these methods are slow, expensive, labor-intensive and require specialized equipment[7]. Thus, effective study of multiple protein expression levels can be complicated, costly and time-consuming. Moreover, these traditional methods of proteomics are not sensitive enough to detect most cytokines (typically at pg/ml concentrations).

Cytokines, broadly defined as secreted cell-cell signaling proteins distinct from classic hormones or neurotransmitters, play important roles in inflammation, innate immunity, apoptosis, angiogenesis, cell growth and differentiation[7]. They are involved in most disease processes, including cancer, obesity and inflammatory and cardiac diseases.

Simultaneous detection of multiple cytokines undoubtedly provides a powerful tool to study cell signaling pathways. Regulation of cellular processes by cytokines is a complex, dynamic process, often involving multiple proteins. Positive and negative feedback loops, pleiotrophic effects and redundant functions, spatial and temporal expression of or synergistic interactions between multiple cytokines, even regulation via release of soluble forms of membrane-bound receptors, all are common mechanisms modulating the effects of cytokine signaling[8-14]. As such, unraveling the role of individual cytokines in physiologic or pathologic processes generally requires consideration and detection of multiple cytokines rather than of a single cytokine.

1. <u>More Data, Same or Less Sample:</u> Antibody arrays provide high-content screening using about the same sample volume as traditional ELISA.

2. <u>Global View of Cytokine Expression:</u> Antibody array screening improves the chances for discovering key factors, disease mechanisms, or biomarkers related to cytokine signaling.

3. <u>Similar (sometimes better) Sensitivity:</u> As little as 4 pg/ml of MCP-1 can be detected using the C-Series array format. In contrast, our similar MCP-1 ELISA assay has a sensitivity of 40 pg/ml of MCP-1.

4. <u>Increased Detection Range:</u> ELISA assays typically detect a concentration range of 100- to 1000-fold. However, RayBiotech arrays can, for example, detect IL-2 at concentrations of 25 to 250,000 pg/ml, a range of 10,000-fold.

5. <u>Better Precision:</u> As determined by densitometry, the inter-array Coefficient of Variation (CV) of spot signal intensities is 5-10%, comparing favorably with ELISA testing (CV = 10-15%).

<u>References</u>

1. Mamlouk O, Balagurumoorthy P, Wang K, Adelstein SJ, Kassis AI. (2012) Bystander effect in tumor cells produced by iodine-125 labeled human lymphocytes. Int J Radiat Biol, 88(12):1019-27.
2. Kocaoemer A, Kern S, Kluter H, Bieback K. (2007) Human AB serum and thrombin-activated platelet-rich plasma are suitable alternatives to fetal calf serum for the expansion of mesenchymal stem cells from adipose tissue. Stem Cells, 25:1270-1278.
3. Ye Z, Lich JD, Moore CB, Duncan JA, Williams KL, Ting JPY. (2008) ATP Binding by Monarch-1/NLRP12 is critical for its inhibitory function. Mol Cell Biol, 28:1841-1850.
4. Sommer G, Kralisch S, Stangl V, Vietzke A, et al. (2009)Secretory products from human adipocytes stimulate proinflammatory cytokine secretion from human endothelial cells. J Cell Biochem, 106(4):729-737.
5. Bouazza B, Kratassiouk G, Gjata B, Perie S, et al. (2009) Analysis of growth factor expression in affected and unaffected muscles of oculo-pharyngeal muscular dystrophy (OPMD) patients: A pilot study. Neuromusc Disorders, 19(3):199-206.
6. Dumortier J, Streblow DN, Moses AV, Jacobs JM, et al.(2008) Human Cytomegalovirus Secretome Contains Factors That Induce Angiogenesis and Wound Healing. J Virol, 82(13):6524-655.
7. Keren Z, Braun-Moscovici Y, Markovits D, Rozin A, Nahir M, et al. (2009) Depletion of B lymphocytes in rheumatoid arthritis patients modifies IL-8-anti-IL-8 autoantibody network. Clin Immunol, 133(1):108-16.
8. Rovin BH, Song H, Hebert LA, Nadasdy T, et al. (2005) Plasma, urine, and renal expression of adiponectin in human systemic lupus erythematosus. Kidney Int, 68:1825-1833.
9. Duncan JA, Gao X, Huang MT-H, O'Connor BP, Thomas CE, et al. (2009) Neisseria gonorrhoeae Activates the Proteinase Cathepsin B to Mediate the Signaling Activities of the NLRP3 and ASC Containing Inflammasome. J Immunol, 182:6460-6469.
10. Pukstadad BS, Ryana L, Floa TH, Stenvika J, et al. (2009) Nonhealing is associated with persistent stimulation of the innate immune response in chronic venous leg ulcers. J Dermatol Sci, 59(2):115-122.
11. Park JE, Tan HS, Datta A, Lai RC, et al. (2010) Hypoxic Tumor Cell Modulates Its Microenvironment to Enhance Angiogenic and Metastatic Potential by Secretion of Proteins and Exosomes. Mol Cell Proteom, 9:1085-1099.
12. Streblow DN, Dumortier J, Moses AV, Orloff SL, Nelson JA. (2008) Mechanisms of Cytomegalovirus Accelerated Vascular Disease: Induction of Paracrine Factors That Promote Angiogenesis and Wound Healing. Curr Top Microbiol Immunol, 325:397-415.
13. Nolting T, Lindecke A, Koutsilie E, Maschke M, et al. (2009) Measurement of soluble inflammatory mediators in cerebrospinal fluid of human immunodeficiency virus-positive patients at distinct stages of infection by solid-phase protein array. J Neruovirol, 15(5-6): 390-400.
14. Pannebaker C, Chandler HL, Nichols JJ. (2010) Tear proteomics in keratoconus. Mol Vision, 16: 1949-1957.

II. How It Works

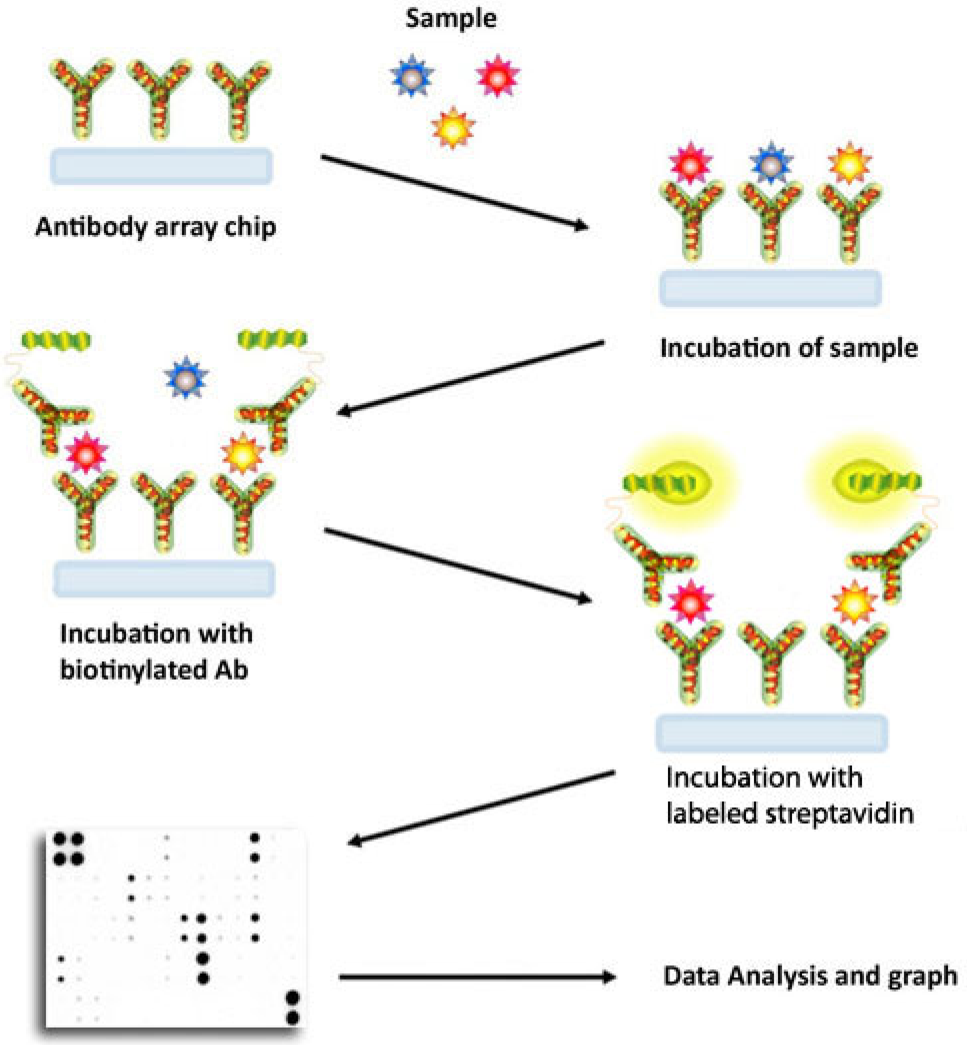

III. Components and Storage

Store kit at ≤ -20°C immediately upon arrival. Kit must used within the 6 month expiration date.

ITEM	COMPONENT	AAH-CYT-5-2	AAH-CYT-5-4	AAH-CYT-5-8	STORAGE TEMPERATURE AFTER THAWING**
1	Antibody Arrays*	2 membranes	4 membranes	8 membranes	≤-20°C
2	Blocking Buffer	1 vial (25 ml)		2 vials (25 ml/ea)	
3	Biotinylated Antibody Cocktail	1 vial	2 vials	4 vials	2-8°C (for up to 3 days after dilution)
4	1,000X HRP-Streptavidin Concentrate	1 vial (50 µl)			2-8 °C
5	20X Wash Buffer I Concentrate	1 vial (10 ml)		1 vial (20 ml)	
6	20X Wash Buffer II Concentrate	1 vial (10 ml)		1 vial (20 ml)	
7	2X Cell Lysis Buffer Concentrate	1 vial (10 ml)		1 vial (16 ml)	
8	Detection Buffer C	1 vial (1.5 ml)		1 vial (2.5 ml)	
9	Detection Buffer D	1 vial (1.5 ml)		1 vial (2.5 ml)	
10	8-Well Incubation Tray w/ Lid	1 tray			Room Temperature
Other Kit Components: Plastic Sheets, Array Map Template, User Manual					

*Each package contains 2 or 4 membranes
**For up to 3 months (unless stated otherwise) or until expiration date

IV. Additional Materials Required

- Pipettors, pipet tips and other common lab consumables
- Orbital shaker or oscillating rocker
- Tissue paper, blotting paper or chromatography paper
- Adhesive tape or plastic wrap
- Distilled or de-ionized water
- A chemiluminescent blot documentation system:
  - CCD Camera
  - X-Ray Film and a suitable film processor
  - Gel documentation system
  - Or other chemiluminescent detection system capable of imaging a western blot

NOTE: *Don't have the time or the equipment to image your membrane? Let the experts at RayBiotech image and analyze your membranes. Contact us for pricing. Telephone: 770-729-2992 Email: techsupport@raybiotech.com*

V. Sample Tips and General Considerations

A. Sample Collection, Preparation and Storage

NOTE: *Optimal methods will need to be determined by each researcher empirically based on researched literature and knowledge of the samples.*

- If not using fresh samples, freeze samples as soon as possible after collection.
- Avoid multiple freeze-thaw cycles. If possible, sub-aliquot samples prior to initial storage.
- Serum-free or low serum containing media (0.2% FBS/FCS) is recommended. If serum containing media is required, testing an uncultured media sample as a negative control is ideal as many types of sera contain cytokines, growth factors and other proteins.
- It is strongly recommended to add a protease inhibitor cocktail to cell and tissue lysate samples.
- Avoid using EDTA as an anti-coagulant for collecting plasma if testing MMPs or other metal-binding proteins.
- Avoid using hemolyzed serum or plasma as this may interfere with protein detection and/or cause a higher than normal background response.
- Avoid sonication of 1 ml or less as this can quickly heat and denature proteins.
- Most samples will not need to be concentrated. If concentration is required, a spin column concentrator with a chilled centrifuge is recommended.
- Always centrifuge the samples hard after thawing (~10,000 RPM for 2-5 minutes) in order to remove any particulates that could interfere with detection.
- ***General tips for preparing serum, plasma, cell culture media, urine, and lysate samples can be viewed on the online Resources page of the website.***

B. Sample Types and Recommended Dilutions/Amounts

NOTE: *Optimal sample dilutions and amounts will need to be determined by each researcher empirically but the below recommendations may be used as a starting point. Blocking Buffer (ITEM 2) should be used to dilute samples. Normalize by loading equal amounts of protein per sample.*

- **Cell Cultured Media:** Neat (no dilution needed)
- **Serum & Plasma:** 2-fold to 10-fold dilution
- **Cell and Tissue Lysates:** Load 50 to 500 µg of total protein (after a 5-fold to 10-fold dilution to minimize the effects of any detergent(s)). Therefore the original lysate concentration should be 1 to 5 mg/ml.
- **Other Bodily Fluids:** Neat or 2-fold to 5-fold dilution

C. Handling Membranes

- The antibody printed side of each membrane is marked by a dash (-) or number (#) in the upper left corner.
- Do not allow membranes to dry out during the experiment or they may become fragile and break OR high and/or uneven background may occur.
- Grasp membranes by the corners or edges only using forceps. DO NOT touch printed antibody spots.

D. Incubations and Washes

- Perform **ALL** incubation and wash steps under gentle rotation or rocking motion (~0.5 to 1 cycle/sec) using an orbital shaker or oscillating rocker to ensure complete and even reagent/sample coverage. Rocking/rotating too vigorously may cause foaming or bubbles to appear on the membrane surface which should be avoided.
- All washes and incubations should be performed in the Incubation Tray (ITEM 10) provided in the kit.
- Cover the Incubation Tray with the lid provided during all incubation steps to avoid evaporation and outside debris contamination.
- Ensure the membranes are completely covered with sufficient sample or reagent volume during each incubation.
- Avoid forceful pipetting directly onto the membrane; instead, gently pipette samples and reagents into a corner of each well.
- Aspirate samples and reagents completely after each step by suctioning off excess liquid with a pipette. Tilting the tray so the liquid moves to a corner and then pipetting is an effective method.

- Optional overnight incubations may be performed for the following step to increase overall spot signal intensities:
  - Sample Incubation
  - Biotinylated Antibody Cocktail Incubation
  - HRP-Streptavidin Incubation

NOTE: *Overnight incubations should be performed at 4°C (also with gentle rocking/shaking). Be aware that longer incubations can also increase the background response so complete liquid removal and washing is critical.*

VI. Chemiluminescence Detection Tips

- Beginning with adding the detection buffers and ending with exposing the membranes should take no more than 10-15 minutes as the chemiluminescent signals may start to fade at this point.
- Trying multiple exposure times is recommended to obtain optimum results.
- A few seconds to a few minutes is the recommended exposure time range, with 30 seconds to 1 minute being suitable for most samples.

Don't have time or the equipment to image your membrane? Let the experts at RayBiotech image and analyze your membranes. Contact us for details and pricing.
Telephone: 770-729-2992
Email: techsupport@raybiotech.com

VII. Component Preparation

NOTE: *Thaw all reagents to room temperature immediately before use. If wash buffers contain visible crystals, warm to room temperature and mix gently until dissolved.*
NOTE: *The Biotinylated Antibody Cocktail (ITEM 3) and the HRP-Streptavidin Concentrate (ITEM 4) vials should be briefly centrifuged (~1000 g) before opening to ensure maximum recovery and mixed well as precipitates may form during storage.*

Item	Component	Preparation	Example
1	Antibody Arrays	No Preparation	N/A
2	Blocking Buffer		
3	Biotinylated Antibody Cocktail*	Pipette 2 ml of Blocking Buffer into each vial. Mix gently with a pipette.	N/A
4	1,000X HRP-Streptavidin Concentrate	Dilute 1,000-fold with Blocking Buffer. Mix gently with a pipette.	10 µl of 1,000X concentrate + 9990 µl of Blocking Buffer = 10 ml of 1X working solution
5	20X Wash Buffer I Concentrate	Dilute **each** 20-fold with distilled or deionized water.	10 ml of 20X concentrate + 190 ml of water = 200 ml of 1X working solution
6	20X Wash Buffer II Concentrate		
7	2X Cell Lysis Buffer Concentrate	Dilute 2-fold with distilled or deionized water.	10 ml of 2X concentrate + 10 ml of water = 20 ml of 1X working solution
8	Detection Buffer C	No Preparation	N/A
9	Detection Buffer D		
10	8-Well Incubation Tray w/ Lid		

*1 vial is enough to test 2 membranes

VIII. Protocol

NOTE: *Prepare all reagents and samples immediately prior to use. See Sections V and VII.* ***ALL*** *incubations and washes must be performed under gentle rotation/rocking (~0.5-1 cycle/sec). Make sure bubbles do not appear on or between the membranes to ensure even incubations.*

1. Remove the kit from storage and allow the components to equilibrate to room temperature.

2. Carefully remove the Antibody Arrays (ITEM 1) from the plastic packaging and place each membrane (printed side up) into a well of the Incubation Tray (ITEM 10). One membrane per well.

NOTE: *The antibody printed side is marked by a dash (-) or number (#) in the upper left corner.*

A. Blocking

3. Pipette 2 ml of Blocking Buffer (ITEM 2) into each well and incubate for 30 minutes at room temperature.

4. Aspirate blocking buffer from each well with a pipette.

B. Sample Incubation

5. Pipette 1 ml of diluted or undiluted sample into each well and incubate for 1.5 to 5 hours at room temperature OR overnight at 4°C.

NOTE: *Longer incubations can help maximize the spot signal intensities. However, doing so can also increase the background response so complete liquid removal and washing is critical.*

6. Aspirate samples from each well with a pipette.

C. First Wash

NOTE: *The 20x Wash Buffer Concentrates I and II (ITEM 5 and 6) must be diluted 20-fold before use. See section VII for details.*

7. Wash Buffer I Wash: Pipette 2 ml of **1X** Wash Buffer I into each well and incubate for 5 minutes at room temperature. Repeat this 2 more times for a total of 3 washes using fresh buffer and aspirating out the buffer completely each time.

8. Wash Buffer II Wash: Pipette 2 ml of **1X** Wash Buffer II into each well and incubate for 5 minutes at room temperature. Reapeat this 1 more time for a total of 2 washes using fresh buffer and aspirating out the buffer completely each time.

D. Biotinylated Antibody Cocktail Incubation

NOTE: *The Biotinylated Antibody Cocktail (ITEM 3) must be prepared before use. See Section VII for details*

9. Pipette 1 ml of the **prepapred** Biotinylated Antibody Cocktail into each well and incubate for 1.5 to 2 hours at room temperature OR overnight at 4°C.

10. Aspirate Biotinylated Antibody Cocktail from each well.

E. Second Wash

11. Wash membranes as directed in Steps 7 and 8.

F. HRP-Streptavidin Incubation

NOTE: *The 1,000X HRP-Streptavidin Concentrate (ITEM 4) must be diluted before use. See section VII for detail.*

12. Pipette 2 ml of **1X** HRP-Streptavidin into each well and incubate for 2 hours at room temperature OR overnight at 4°C.

13. Aspirate HRP-Streptavidin from each well.

G. Third Wash

14. Wash membranes as directed in Steps 7 and 8.

H. Chemiluminescence Detection

NOTE: *Do not allow membranes to dry out during detection.*

15. Transfer the membranes, printed side up, onto a sheet of chromatography paper, tissue paper, or blotting paper lying on a flat surface (such as a benchtop).

16. Remove any excess wash buffer by blotting the membrane edges with another piece of paper.

17. Transfer and place the membranes, printed side up, onto a plastic sheet (provided) lying on a flat surface.

NOTE: *Multiple membranes can be placed next to each other and fit onto a single plastic sheet. Use additional plastics sheets if necessary.*

18. Into a single clean tube, pipette equal volumes (1:1) of Detection Buffer C (ITEM 8) and Detection Buffer D (ITEM 9). Mix well with a pipette.

EXAMPLE: *250 µl of Detection Buffer C + 250 µl of Detection Buffer D = 500 µl (enough for 1 membrane)*

19. Gently pipette 500 µl of the Detection Buffer mixture onto each membrane and incubate for 2 minutes at room temperature (DO NOT ROCK OR SHAKE). <u>Immediately afterwards, proceed to Step 20.</u>

NOTE: *Exposure should ideally start within 5 minutes after finishing Step 19 and completed within 10-15 minutes as chemiluminescence signals will fade over time. If necessary, the signals can usually be restored by repeating washing, HRP-Streptavidin and Detection Buffers incubations (Steps 11-19).*

20. Place another plastic sheet on top of the membranes by starting at one end and gently "rolling" the flexible plastic sheet across the surface to the opposite end to smooth out any air bubbles. The membranes should now be "sandwiched" between two plastic sheets.

NOTE: *Avoid "sliding" the top plastic sheet along the membranes' printed surface.* *If using X-ray film, do not use a top plastic sheet so that the membranes can be directly exposed to the film.*

21. Transfer the sandwiched membranes to the chemiluminescence imaging system such as a CCD camera (recommended) and expose.

NOTE: *Optimal exposure times will vary so performing multiple exposure times is strongly recommended. See Section VI for additional details.*

I. Storage

22. To store, without direct pressure, gently sandwich the membranes between 2 plastic sheets (if not already), tape the sheets together or use plastic wrap to secure them, and store at $\leq$ -20°C for future reference.

IX. Typical Results

Typical Results obtained with RayBio C-series Antibody Arrays

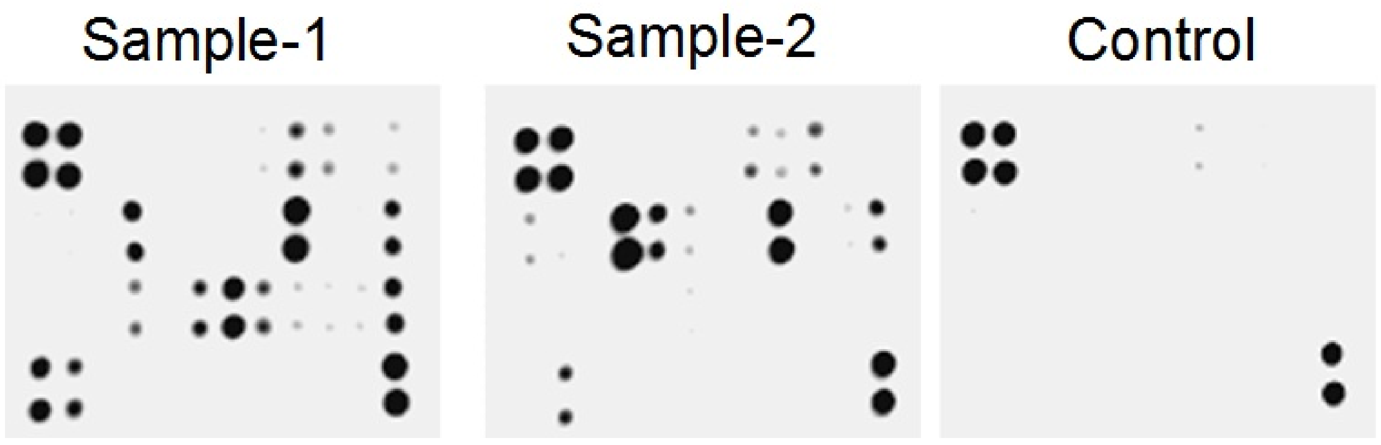

The preceding figures present typical images obtained with RayBio® C-Series Antibody Arrays. These membranes were probed with conditioned media from two different cell lines. Membranes were exposed with UVP Bioimaging Epichem 3 Darkroom for 1 minute.

Note the strong signals of the Positive Control Spots in the upper left and lower right

corners. (See below for further details on the control spots.)

The signal intensity for each antigen-specific antibody spot is proportional to the relative concentration of the antigen in that sample. Comparison of signal intensities for individual antigen-specific antibody spots between and among array images can be used to determine relative differences in expression levels of each analyte sample-to-sample or group-to-group.

X. Interpreting the Results

A. Control Spots

Positive Control Spots (POS) - Controlled amount of biotinylated antibody printed onto the array. Used for normalization and to orientate the arrays.
Negative Control Spots (NEG) - Buffer printed (no antibodies) used to measure the baseline responses. Used for determining the level of non-specific binding of the samples.
Blank Spots (BLANK) - Nothing is printed here. Used to measure the background response.

B. Data Extraction

Visual comparison of array images may be sufficient to see differences in relative protein expression. However, most researchers will want to perform numerical comparisons of the signal intensities (or more precisely, signal densities), using 2-D densitometry. Gel/Blot documentation systems and other chemiluminescent or phosphorescent detection systems are usually sold as a package with compatible densitometry software.

Any densitometry software should be sufficient to obtain spot signal densities from your scanned images. One such software program, ImageJ, is available for free from the NIH website along with an array plug-in.

We suggest using the following guidelines when extracting densitometry data from our array images:

- For each array membrane, identify a single exposure that the exhibits a high signal to noise ratio (strong spot signals and low background response). Strong Positive Control Spot signals but not too strong that they are "bleeding" into one

another is ideal. The exposure time does not need to be identical for each array, but Positive Control signals on each array image should have similar intensities.

- Measure the density of each spot using a circle that is roughly the size of one of the largest spots. Be sure to use the same extraction circle dimensions (area, size, and shape) for measuring the signal densities on every array for which you wish to compare the results.
- For each spot, use the summed signal density across the entire circle (ie, total signal density per unit area).

C. Data Analysis

NOTE: *RayBiotech offers Microsoft® Excel-based Analysis Software Tools for each array kit for automatic analysis. Please visit the website at www.raybiotech.com or contact us for ordering information.*

Once the raw numerical densitometry data is extracted, the background must be subtracted and the data normalized to the Positive Control signals to analyze.

Background Subtraction: Select values which you believe best represent the background. If the background is fairly even throughout the membrane, the Negative Control Spots (NEG) and/or Blank Spots (BLANK) should be similar and are accurate for this purpose.

Positive Control Normalization: The amount of biotinylated antibody printed for each Positive Control Spot is consistent from array to array. As such, the intensity of these Positive Control signals can be used to normalize signal responses for comparison of results across multiple arrays, much like housekeeping genes and proteins are used to normalize results of PCR gels and Western Blots, respectively.

To normalize array data, one array is defined as "Reference Array" to which the other arrays are normalized to. The choice of the Reference Array is arbitrary.

NOTE: *The RayBio® Analysis Software Tools always designate Array 1/Sample 1 as the Reference Array.*

Next, the simple algorithm below can be used to calculate and determine the signal fold expression between like analytes.

X(Ny) = X(y) * P1/P(y)

Where:
P1=mean signal density of Positive Control spots on reference array
P(y)=mean signal density of Positive Control spots on Array "y"
X(y)= X(Ny)=normalized signal intensity for spot "X" on Array "y"

For example:

Let's determine the relative expression of IL-6 on two different arrays (Arrays 1 and 2). Let's assume that the duplicate signals for the IL-6 spots on each array are identical (or that the signal intensity used in the following calculation is the mean of the two duplicates spots). Also assume the following:

P1 = 2500
P2 = 2700
IL-6 (1) = 300
IL-6 (2) = 455

Then IL-6(N2) = 455 *2500/2700 = 421.30
The fold increase of IL-6(N2) vs IL-6(1) = 421.3/300 = 1.40-fold increase or a 40% increase in the signal intensity of IL-6 in Array 2 vs. Array 1.

XI. Array Map

	A	B	C	D	E	F	G	H	I	J	K
1	POS	POS	POS	POS	NEG	NEG	ENA-78 (CXCL5)	G-CSF	GM-CSF	GRO a/b/g	GRO alpha (CXCL1)
2	I-309 (CCL1)	IL-1 alpha (IL-1 F1)	IL-1 beta (IL-1 F2)	IL-2	IL-3	IL-4	IL-5	IL-6	IL-7	IL-8 (CXCL8)	IL-10
3	IL-12 p40/p70	IL-13	IL-15	IFN-gamma	MCP-1 (CCL2)	MCP-2 (CCL8)	MCP-3 (CCL7)	M-CSF	MDC (CCL22)	MIG (CXCL9)	MIP-1 beta (CCL4)
4	MIP-1 delta	RANTES (CCL5)	SCF	SDF-1 alpha	TARC (CCL17)	TGF beta 1	TNF alpha	TNF beta (TNFSF1B)	EGF	IGF-1	Angiogenin
5	OSM	TPO	VEGF-A	PDGF-BB	Leptin	BDNF	BLC (CXCL13)	Ck beta 8-1 (CCL23)	Eotaxin-1 (CCL11)	Eotaxin-2 (CCL24)	Eotaxin-3 (CCL26)
6	FGF-4	FGF-6	FGF-7 (KGF)	FGF-9	FLT-3 Ligand	Fractalkine (CX3CL1)	GCP-2 (CXCL6)	GDNF	HGF	IGFBP-1	IGFBP-2
7	IGFBP-3	IGFBP-4	IL-16	IP-10 (CXCL10)	LIF	LIGHT (TNFSF14)	MCP-4 (CCL13)	MIF	MIP-3 alpha	NAP-2 (CXCL7)	NT-3
8	NT-4	OPN (SPP1)	OPG (TNFRSF11	PARC	PLGF	TGF beta 2	TGF beta 3	TIMP-1	TIMP-2	POS	POS

XII. Troubleshooting Guide

Problem	Cause	Recommendation
**No Signals (not even positive control spots)**	Chemiluminescent imager is not working properly	Contact imager manufacturer
	Too Short Exposure	Expose the membranes longer
	Degradation of components due to imporper storage	Store entire kit at $\leq$ -20°C. Do not use kit after expiration date. See storage guidelines.
	Improper preparation or dilution of the HRP-Streptavidin	Centrifuge vial briefly before use, mix well, and do not dilute more than 1000-fold
	Waiting too long before exposing	The entire detection process should be completed in 10-15 minutes
**Signals from Positive Control Spots visible, but no other spots visible**	Low sample protein levels	Decrease sample dilution, concentrate samples, or load more protein initially
	Skipped Sample Incubation Step	Samples must be loaded after the blocking step
	Incubations Too Short	Ensure the incubations are performed for the appropriate time or try the optional overnight incubation(s)
**Uneven Signals and/or Background**	Bubbles present on or below membrane	Don't rock/rotate the tray too vigorously or pipette the sample or reagent with excessive force
	Insufficient sample or reagent volume	Load enough sample and reagent to completely cover the membrane
	Insufficient mixing of reagents	Gently mix all reagents before loading onto the membrane, especially the HRP-Streptavidin and Biotin Antibody Cocktail
	Rocking/Rotating on an uneven surface while incubating	Rock/rotate on a flat surface or the sample or reagent can "pool" to one side
**High Background Signals or all Spots Visible**	Too much HRP-Streptavidin or Biotinylated Antibody Cocktail	Prepare these signal enhancing components precisely as instructed
	Prepare these signal enhancing components precisely as instructed	Do not let the membranes dry out during the experiment. Cover the incubation tray with the lid to minimize evaporation
	Too High of Sample Protein Concentration	Increase dilution of the sample or load less protein
	Exposed Too Long	Decrease exposure time
	Insufficient Washing	Ensure all the wash steps are carried out and the wash buffer is removed completely after each wash step
	Non-specific binding	Ensure the blocking buffer is stored and used properly

RayBio® C-Series: Membrane-Based Antibody Arrays

A variety of C-Series array kits are available, detecting anywhere from 10 to 274 proteins simultaneously, visit http://www.raybiotech.com/c-series-membrane-based-antibody-arrays.html for details.

This product is for research use only.

©2015 RayBiotech, Inc
